# Transition Metal-Based Oxidation Catalysts to Mitigate Methane Emissions from Low-Concentration Sources

**DOI:** 10.1021/acsomega.5c10282

**Published:** 2026-04-28

**Authors:** Nardana Bazybek, Luigi Vicidomini, Efthymios Kantarelis, Klas Engvall, Shareq Mohd Nazir

**Affiliations:** † 7655 KTH Royal Institute of Technology, Department of Chemical Engineering, Stockholm, SE 114 28, Sweden; ‡ Politecnico Milano, Department of Chemical Engineering, Milan, IT 20133, Italy

## Abstract

Methane is a greenhouse gas 28 times more potent than CO_2_, with wetlands and agricultural practices accounting for 32 and 38% of total natural and anthropogenic methane emissions, respectively. The diluted and distributed nature of these emissions makes them hard to mitigate. Therefore, effective mitigation techniques are essential to reduce its impact, with catalytic oxidation emerging as a promising solution. In this study, methane oxidation over transition metal oxides is investigated to remove low-concentration CH_4_ due to its high efficiency and low secondary pollution. The catalytic performance of different transition metal oxides (Co, Ni, and Mn), including single and binary metal oxides, was investigated under controlled conditions to facilitate direct comparison. The experimental investigation revealed that Co_3_O_4_–Mn_*x*_O_*y*_ and Co_3_O_4_ catalysts achieved a 90% methane conversion rate at 330 and 380 °C. The high surface area and small crystallite size of Co_3_O_4_–Mn_*x*_O_*y*_ increase the exposure of active sites, while the synergistic effect of Mn and Co promotes oxygen vacancy formation. In contrast, Co_3_O_4_ benefits from a high density of surface oxygen vacancies and an optimal acid–base balance, enabling intermediate stabilization and rapid redox cycling. By linking catalytic performance to structural features such as surface morphology, oxygen vacancies, acidity–basicity, and crystallite structures, this study provides insights into the reaction mechanisms governing methane oxidation.

## Graphical abstract

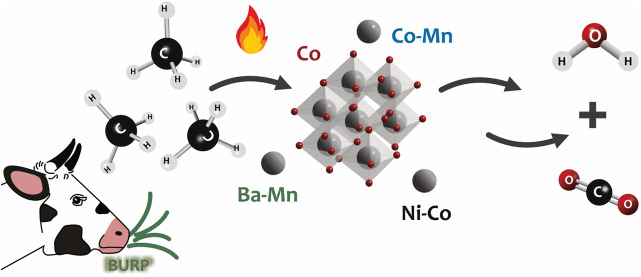

## Introduction

Methane is one of the major greenhouse gases driving global warming. Despite its lower atmospheric concentration compared to carbon dioxide, methane exhibits a higher efficiency in absorbing thermal infrared radiation, resulting in a greater global warming potential (GWP).^1^ Over a 20-year period, methane is 84 times more potent than carbon dioxide and 28 times more potent over a 100-year period.^2^

Anthropogenic methane emissions account for approximately two-thirds of total global methane emissions. Agricultural activities are the leading contributors to these emissions, mainly due to cattle, sheep, and other ruminants, as well as rice cultivation and the management of manure and waste.^3^ These emissions are on the rise, propelled by the global increase in both overall and per capita meat consumption as the world’s population and wealth expand.^4^ Following the COP29 summit, 159 countries committed to reducing methane emissions by 30% by 2030. This commitment has spurred the development of technologies aimed at reducing emissions and removing methane from the atmosphere.^5^

One of the most promising and cost-effective methods for treating low-concentration methane is oxidation, which offers several advantages, including high efficiency, minimal secondary pollution, and high oxidation rates.^6,7^ However, methane oxidation remains challenging due to the strong C–H bond (bond energy approximately of 439 kJ/mol) and the nonpolar nature of the CH_4_ molecule.^8^ While oxidation is exothermic, releasing 890 kJ/mol upon full oxidation, the energy released is insufficient to self-sustain the reaction at low methane concentrations (0.1 vol % for a reverse flow reactor and 0.4 vol % for a catalytic monolith reactor).^9,10^ This limited energy release from the exothermic reaction causes only a slight increase in the gas mixture temperature, with an insignificant effect on the surrounding environment.^10^ As a result, one of the main challenges in low-concentration methane conversion is a high light-off temperature required for sustained oxidation reactions.

Noble and transition metal oxide catalysts have the potential to address these limitations by lowering the activation energy from its original range (typically 312–450 kJ/mol in the uncatalyzed reaction) to 30–42 kJ/mol.^11,12^ Noble metal catalysts (Pd, Pt, Rh, and Au) are characterized by their low light-off temperatures, which are based on their ability to activate the C–H bond and O–O bonds to generate free radicals and initiate chain reactions.^13^ However, their high cost and susceptibility to CO poisoning at low temperatures have driven the search for more affordable alternatives.^14,15^

Transition metal oxide (TMO) catalysts are gaining attention for their redox properties, oxygen storage capacity, and thermal stability. Transition metals with multiple valence states play an important role in the catalytic process by facilitating redox cycles that alternate between high and low oxidation states, effectively restoring and releasing lattice oxygen during the reaction.^16^ In some instances, TMO-based catalysts have demonstrated performance comparable to that of the noble-metal-based systems.^13,17^ For instance, Setiawan et al.^18^ compared the performance of bulk Co_3_O_4_ and Fe_2_O_3_ with 0.6% CH_4_ balanced in air, revealing a T_50_ of 350 °C for Co_3_O_4_ versus 440 °C for Fe_2_O_3_. Oxygen temperature-programmed desorption (O_2_-TPD) revealed that Co_3_O_4_ exhibits stronger oxygen adsorption and higher surface oxygen coverage than Fe_2_O_3_, directly correlating with its lower light-off temperature. Similarly, in the MnO_2_ system, Xu et al.^19^ found that among α-, γ-, and δ-MnO_2_ catalysts, the α-phase exhibited better activity (T_50_ = 400 °C, with a gas mixture of 1 vol % CH_4_, 20% O_2_ and Ar balance) due to its abundant Mn–O–Mn bridge-type oxygen. These sites demonstrated the lowest CH_4_ adsorption energy while effectively activating O_2_ to form reactive Mn=O species. Mixed TMO catalysts exhibit enhanced catalytic activity relative to single metal oxides because of their unique cation properties and high oxygen vacancy density, positioning them as promising candidates for methane oxidation.^16^ In an experiment utilizing a mixture of 10% CH_4_ balanced in Ar and O_2_ with the NiCo_2_O_4_ catalyst, higher methane conversion was achieved compared to pure Co_3_O_4_ or NiO with similar spinel structures and surface areas, achieving complete oxidation at 350 °C. This enhanced performance is attributed to the benefits of Ni doping, which is more effective in facilitating the C–H bond cleavage. Akbari et al.^20^ demonstrated that incorporating metals into MnO_*x*_ matrices changes methane oxidation pathways, with CuO-MnO_*x*_ achieving the lowest light-off temperature (T_50_ = 355 °C, gas mixture: O_2_/CH_4_ = 6:1) due to its elevated surface area and enhanced reducibility and synergistic Cu–Mn interactions.

Despite extensive studies on TMO catalysts for methane oxidation, systematic comparisons of distinct TMO systems remain limited. Most published work consists of reviewing articles summarizing trends in the field, and even experimental research largely focuses on individual metal oxides or their supports, rather than comparative analysis of mixed or diverse TMO combinations. Additionally, different experimental conditions, including preparation methods, reactor designs, methane inlet concentrations, and space velocities, complicate direct cross-study comparisons, making it difficult to draw conclusions about catalytic performance. For instance, Parades et al. established the following order of reactivity: Co_3_O_4_ > Mn_2_O_3_ > Cr_2_O_3_ > CuO > NiO. While these trends are attributed to the presence of reticular oxides, high methane adsorption, and surface areas,^21^ such properties alone might be insufficient to explain the underlying mechanism or the synergistic interactions between TMO without correlating them to each other and with catalytic performance.

This lack of standardized data presents a barrier, as developing new processes for the thermal catalytic oxidation of methane is particularly challenging when the reactor designs and reaction conditions presented in literature are different for different catalysts. Therefore, this work aims to provide a comparison of four distinct bulk TMO catalysts: a benchmark (Co_3_O_4_), a redox-synergy composite (Co_3_O_4_–Mn_*x*_O_*y*_), an alkaline-promoted system (Mn_*x*_O_*y*_–BaO), and a spinel (NiCo_2_O_4_). By synthesizing, characterizing, and testing these functionally diverse materials under identical conditions, the ambiguities of literature comparisons are eliminated, allowing for a focused investigation to understand the synergistic effects of transition metals. For this, it seeks to elucidate structure–activity relationships by examining how factors such as morphology, surface acidity/basicity, oxygen speciation, and redox states influence catalytic performance in relation to the underlying reaction mechanisms. This research advances scientific literature by providing insights into the rational design of TMO-based catalysts for the low-concentration and low-temperature catalytic oxidation of methane, which will result in designing energy-efficient methane mitigation processes.

## Experimental Section

### Catalyst Synthesis

#### Synthesis of Co_3_O_4_

The Co_3_O_4_ catalyst was synthesized using the coprecipitation method. Cobalt chloride hexahydrate (CoCl_2_·6H_2_O), used as the precursor, was dissolved in deionized water and stirred magnetically for 30 min. A 1 M sodium carbonate (Na_2_CO_3_) solution was then added to the mixture, resulting in light purple precipitates, which were continuously stirred at 60 °C for 5 h. Following precipitation, the product was washed multiple times with water until a neutral pH was achieved. The collected precipitates were dried overnight at 80 °C and subsequently calcined at 500 °C for 5 h, completing the synthesis process.

#### Synthesis of Co_3_O_4_–Mn_*x*_O_*y*_

The catalyst Co_3_O_4_–Mn_*x*_O_*y*_ (Co:Mn = 5:1) was synthesized via the coprecipitation method. Cobalt nitrate hexahydrate (Co­(NO_3_)_2_·6H_2_O) and manganese acetate tetrahydrate (Mn­(CH_3_COO)_2_·4H_2_O) were used as precursors, each of which was dissolved separately in 15 mL of deionized water under magnetic stirring before mixing. A 0.1 M ammonium bicarbonate (NH_4_HCO_3_) solution was then added dropwise as the precipitating agent until the pH reached 9–10, resulting in the formation of a white precipitate. The mixture was stirred at room temperature for 8 h, followed by washing with deionized water until the pH stabilized at 7. The resulting precipitate was air-dried overnight at room temperature, further dried at 90 °C for 6 h, and finally calcined at 400 °C for 4 h.

#### Synthesis of Mn_*x*_O_*y*_–BaO

Catalyst containing Mn_*x*_O_*y*_ and BaO (Mn:Ba = 15:1) was synthesized using a solid-state mechanochemical reaction involving manganese nitrate tetrahydrate (Mn­(NO_3_)_2_·4H_2_O), barium nitrate (Ba­(NO_3_)_2_), and ammonium carbonate ((NH_4_)_2_CO_3_).^22^ The preparation process entailed manually mixing and grinding the specified amounts of metal salt precursors and ammonium carbonate in an agate mortar for 20 min at room temperature. During this mixing process, water from the metal salt precursors was released, forming a wet cake mixture. The wet cake was then dried overnight at 100 °C, followed by calcination at 500 °C for 5 h.

#### Synthesis of NiCo_2_O_4_

The catalyst NiCo_2_O_4_ was prepared by the following procedure: 2.37 g of CoCl_2_·6H_2_O, 1.19 g of nickel chloride hexahydrate (NiCl_2_·6H_2_O) and 2.7 g of urea (CH_4_N_2_O) were dissolved in distilled water and stirred for approximately 10 min until a transparent, pink-colored solution was obtained. The resulting reacting mixture was then transferred to the stainless-steel autoclave and subjected to hydrothermal treatment at 120 °C for 6 h. Subsequently, the autoclave was cooled to room temperature, then the mixture underwent filtration and washing with distilled water and ethanol until a neutral pH was reached. The washed material was then dried at 80 °C in a furnace overnight. Finally, the synthesized material underwent calcination at 500 °C for 4 h.

### Catalyst Characterization

The crystal structure of the catalyst was examined through X-ray diffraction (XRD) using the PANalytical X’Pert Pro with a Cu–Kα radiation source, operating at 40 kV and 40 mA. The samples underwent scanning at intervals of 0.02 deg/step within the 2θ range of 5–80 degrees, with a scan time of 2 s per step. The diffraction patterns were identified by matching them with the data provided in the Joint Committee on Powder Diffraction Standards (JCPDS) cards from the International Center for Diffraction Data (ICDD), enabling the identification of crystallite phases. The crystallite size was calculated using the Scherrer equation after performing peak fitting and extracting the full width at half-maximum (fwhm):

1 $$D=\frac{K\lambda }{\beta \cos\theta }$$

In eq 1, *K* is the shape factor (0.9), λ is the X-ray wavelength (1.54 Å), β is the corrected fwhm in radians, and θ is the Bragg angle. The Brunauer–Emmett–Teller (BET) specific surface areas were determined through adsorption–desorption isotherms using a Micromeritics ASAP 2010 system with N_2_ as the sorbate at −196 °C. Prior to surface area measurement, all the samples were degassed at 300 °C, and pore size distributions were calculated from the desorption isotherm employing the Barrett–Joyner–Halenda (BJH) model. The surface chemical composition was analyzed using the Kratos AXIS Supra+ X-ray photoelectron spectrometer (XPS) with an Al–Kα source. The analysis was performed to determine the elemental composition and oxidation states of the metals present on the catalyst surface before and after the reaction, providing insights into the chemical environment and potential changes in surface properties. Binding energy (BE) values were calibrated using the C 1s peak of adventitious carbon at 284.8 eV as a reference. Details of the peak-fitting procedure are described in the Supporting Information. In addition, scanning electron microscopy (SEM) images were obtained using a Thermo Fisher Scientific Apreo 2s LoVac microscope equipped with a field emission gun source to examine the surface morphology and particle size distribution. An Ultradry 60 mm^2^ energy-dispersive X-ray spectroscopy (EDS) detector was employed, enabling integrated EDS mapping through the ChemiSEM feature within the SEM software to analyze the elemental composition and distribution. Temperature-programmed desorption of NH_3_ (NH_3_-TPD) and CO_2_ (CO_2_-TPD) experiments were performed using Micromeritics Autochem 2910 to analyze the acidity/basicity properties. For both analyses, a sample mass of 0.11 g was used. In NH_3_-TPD, the catalyst was saturated with 5% NH_3_/He gas at 200 °C, followed by He-purged heating from 200 to 800 °C (for Mn_*x*_O_*y*_–BaO) or 900 °C (for Co_3_O_4_) at a heating rate of 5 °C/min to obtain the NH_3_ desorption profile. Similarly, CO_2_-TPD involved adsorbing 5% CO_2_/Ar gas onto the catalyst under analogous conditions, with subsequent thermal desorption profiling in an Ar flow. Both experiments utilized identical heating protocols to assess acid and base site distributions, respectively.

### Catalytic Activity Tests

The catalytic performance of the TMO catalysts was assessed in a laboratory-scale stainless-steel fixed-bed reactor with an internal diameter of 8 mm. The reactor was positioned vertically within a PID-controlled electric furnace with three independently controlled heating zones. A temperature ramp of 10 °C/min was achieved by controllers that regulated the furnace temperature. The thermocouple was placed right above the catalyst bed to monitor the temperature. A schematic illustration of the experimental setup is presented in Figure S1. The measurements were carried out across a range of temperatures (one measurement/s), starting from 200 °C, with the final temperatures set at 350 °C for Co_3_O_4_–Mn_*x*_O_*y*_, 400 °C for Co_3_O_4_ and 480 °C for the remaining catalysts. Prior to testing, the catalyst was sieved to a particle size fraction of 50–200 μm to minimize mass transfer limitations and packed to a bed length of approximately 1.5 cm. The gas hourly space velocity (GHSV) was set at 24000 mL g^–1^ h^–1^ (NTP) to ensure consistent testing conditions for all catalysts, minimizing variations in residence time, so that observed differences in catalytic performance are due to the catalyst’s intrinsic properties rather than operational factors, which was ensured by Weisz-Prater and Mears criteria calculation. The feed composition consisted of 1% CH_4_ balanced in air (20% O_2_ + 79% N_2_). The catalyst activity was evaluated by the light-off temperature (T_50_), which represents the temperature at which a CH_4_ conversion of 50% was obtained. The reliability of the fixed-bed reactor was evaluated by conducting the blank test with identical experimental conditions and replacing the catalyst with silica support material. No CH_4_ conversion was observed in the temperature range of 25–550 °C.

The heated flame ionization detector (HFID) is used to measure the concentration of effluent gas composition online. The HFID measures samples on a wet basis, with all components in contact with the sample heated and precisely controlled at 190 °C. The use of HFID is sufficient since there are no other combustible gases in the mixture, and the amount of O_2_ is high enough to prevent the formation of intermediates. Additionally, the resulting gas composition is confirmed using micro-GC. The methane conversion of the catalyst is calculated using the following formula:

2 $$X_{CH_{4}\%}=\frac{C_{CH_{4in}}-C_{CH_{4out}}}{C_{CH_{4in}}}\times 100$$

In eq 2, *C*_CH4in_ represents the initial concentration of methane in the feed, while *C*_CH4out_ denotes the final concentration of CH_4_ in the product stream. All concentrations are expressed in volume percent (vol %). Additionally, to evaluate the intrinsic activity of the catalyst, the methane conversion was normalized by the BET surface area. The moles of methane converted were calculated from the inlet flow rate and conversion data, then divided by the catalyst’s surface area (m^2^/g) and catalyst mass (g) to determine activity per unit surface area (μmol/m^2^).

The apparent activation energy (*E*_a_) and pre-exponential factor (*A*) were determined from temperature-dependent kinetic measurements, where the methane conversion ranged between 10 and 20%. The apparent rate constant (*k*) was calculated assuming first-order kinetics with respect to methane partial pressure, as the reaction rate was proportional to the pressure of the methane in preliminary experiments:

3 $$k=\frac{F_{in}\times P_{CH_{4in}}}{W\times P_{std}}\times \left[\ln\frac{1}{1-X}\right]$$

where *F*_in_ is the inlet molar flow rate (mol/s), *W* is the catalyst mass (g), and *P*_CH4in_ and *P*_std_ are the inlet methane partial pressure (Pa) and the pressure under standard conditions, respectively. The activation energy (*E*_a_) and pre-exponential factor (*A*) were obtained from the Arrhenius plot of the ln­(*k*) vs 1/*T*:

4 $$\ln k=\ln A-\frac{E_{a}}{R}\times \frac{1}{T}$$

All kinetic data were derived from the low-conversion region to minimize mass- and heat-transfer effects and ensure operation within the kinetic regime.

## Results and Discussion

### Catalytic Performance

Figure 1A shows methane conversion as a function of reaction temperature over the prepared catalysts. The results indicate that catalytic activity was significantly affected by the catalyst composition. According to the light-off temperatures (T_50_) listed in Table 1, the introduction of Mn into Co_3_O_4_ resulted in a slight improvement in activity, reducing the T_50_ from 332 °C for pure Co_3_O_4_ to 314 °C for the Co_3_O_4_–Mn_*x*_O_*y*_ catalyst. Interestingly, while Co_3_O_4_–Mn_*x*_O_*y*_ exhibits a higher activation energy (91.05 kJ/mol) than Co_3_O_4_, its improved light-off performance is driven by a high pre-exponential factor (*A* = 3.29 × 10^7^ mol/(g·s)). This suggests that the total number of accessible active sites compensates for the higher energy barrier per site.^23^ This high site density can be attributed to the increased surface area and small crystallite size, as demonstrated by XRD and N_2_ physisorption. Additionally, the increase in Co^3^+^^/Co^2+^ suggests that the manganese promotes the stabilization of higher cobalt oxidation states, which facilitates the redox cycling.^24^ In comparison, Co_3_O_4_ demonstrated good catalytic properties (T_50_ of 332 °C) and exhibited the lowest activation energy among all catalysts, at 71.23 kJ/mol. This indicates that the active sites on Co_3_O_4_ are intrinsically more reactive per site, which can be explained by its redox flexibility (Co^3^+^^/Co^2+^ transitions) and the presence of abundant surface oxygen vacancies (from XPS analysis) that facilitate reactant adsorption and activation.^14,25^ Mn_*x*_O_*y*_–BaO demonstrated moderate catalytic activity with a T_50_ around 372 °C, while exhibiting activation energy of 103.78 kJ/mol and its relatively low pre-exponential factor (*A* = 6.16 × 10^2^ mol/(g·s)). This can be partially attributed to the incorporation of Ba to enhance the basic properties of the catalyst, as can be seen from the CO_2_-TPD result. However, the significantly larger ionic radius of barium, nearly twice that of manganese, introduces lattice strain, which can hinder the optimal structural arrangement needed for high catalytic performance. Lastly, NiCo_2_O_4_ exhibited the lowest catalytic performance, with a light-off temperature of 436 °C and achieving T_90_ at 475 °C. This performance is reflected in its high activation energy of 122.02 kJ/mol and relatively low pre-exponential factor (*A* = 2.23 × 10^3^ mol/(g·s)) compared to other cobalt-based catalysts. Notably, this performance differs from that reported in previous studies,^26^ where NiCo_2_O_4_ achieved T_90_ at approximately 300 °C. Such discrepancies can be attributed to differences in synthesis methodology, which can lead to variations in physicochemical properties, including crystallite size, surface area, and surface oxygen characteristics, all of which influence methane oxidation activity.

**Figure 1:**
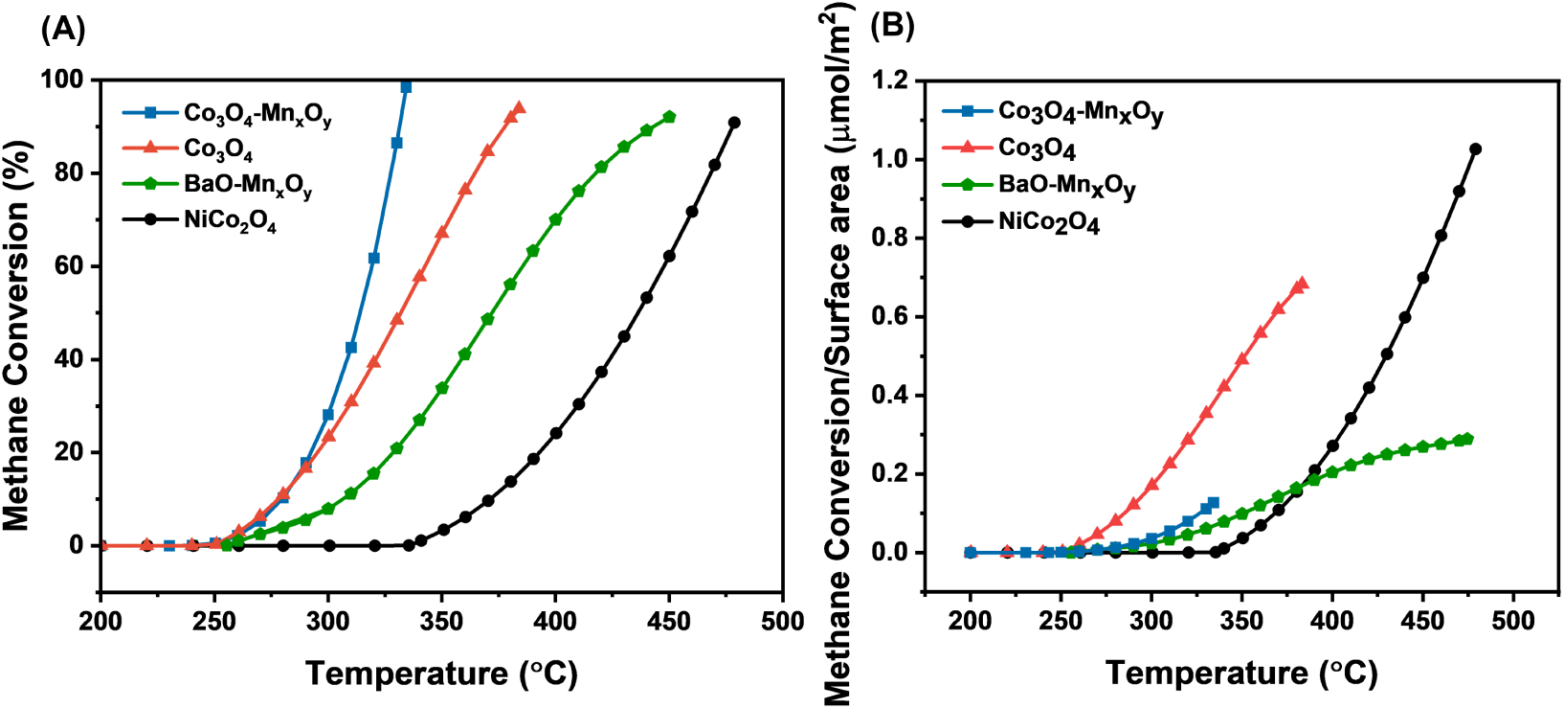
(A) CH_4_ conversion vs temperature for bulk TMO catalysts. (B) Surface-area-normalized number of moles of CH_4_ reacted to compare intrinsic catalytic activity.

**Table 1:** Catalytic Activity and Arrhenius Law Parameters of Prepared Catalysts

Catalyst	T_50_ (°C)	T_90_ (°C)	*E*_a_ (kJ/mol)	*A* (mol/g·s)
Co_3_O_4_–Mn_*x*_O_*y*_	314	330	91.05	3.29 × 10^7^
Co_3_O_4_	332	380	71.23	6.64 × 10^3^
Mn_*x*_O_*y*_–BaO	372	440	103.78	6.16 × 10^2^
NiCo_2_O_4_	436	475	122.02	2.23 × 10^3^

When the catalytic performance was normalized by the specific surface area (*S*_BET_), the intrinsic properties of the catalysts became more evident, as shown in Figure 1B, with Co_3_O_4_ exhibiting higher normalized activity than Co_3_O_4_–Mn_*x*_O_*y*_. This change suggests that the high *S*_BET_ of Co_3_O_4_–Mn_*x*_O_*y*_ contributed substantially to its catalytic performance due to the surface area, which provides more easily accessible active sites,^27^ and enhanced oxygen mobility,^28^ which are critical for efficient methane oxidation. Notably, Co_3_O_4_ emerged as the intrinsically most active catalyst, aligning with its lowest activation energy. In contrast, NiCo_2_O_4_ showed the lowest intrinsic activity, which is consistent with its higher light-off temperature observed.

To further position the present result within the literature, a comparison with previously reported bulk transition metal oxide catalysts for methane oxidation is provided in Supporting Information (Table S1). It is important to note that substantial variations in testing conditions exist across reported studies, which complicate direct performance comparison. This variability emphasizes the importance of establishing clear structure–performance relationships under controlled experimental conditions. Within this framework, the Co_3_O_4_–Mn_*x*_O_*y*_ catalyst exhibits competitive performance in terms of T_90_ relative to reported bulk TMO systems (Table S1).

### Catalyst Characterization

The morphology of the TMO samples was characterized by SEM. As shown in Figure 2, all four compounds have different particle sizes and morphologies due to the different synthesis methodologies. Coprecipitation (Co_3_O_4_, Co_3_O_4_–Mn_*x*_O_*y*_) leads to fine, homogeneously distributed particles, while the solid-state mechanochemical method (Mn_*x*_O_*y*_–BaO) results in larger, highly crystalline structures.^29^ In contrast, the hydrothermal method (NiCo_2_O_4_) facilitates controlled crystal growth, yielding anisotropic morphology due to temperature and pressure.^30^ Co_3_O_4_ exhibits a regular, rigid polyhedral structure with the lowest porosity (0.166 cm^3^ g^–1^), while the Co_3_O_4_–Mn_*x*_O_*y*_ mixture displays a dendrite-like morphology. Despite both materials being synthesized via the coprecipitation method, SEM images show that the particle size of the Co_3_O_4_–Mn_*x*_O_*y*_ mixture (1–10 μm) is significantly smaller than that of Co_3_O_4_ (10–100 μm). As depicted in Figure 2D the synthesized NiCo_2_O_4_, produced via the hydrothermal method, consists of numerous microspheres with diameters ranging from approximately 3 to 6 μm. Each microsphere is composed of radially oriented nanowires that self-organize into a shell-like structure, giving rise to a distinctive sea-urchin-like morphology. Despite the morphology, the surface area of NiCo_2_O_4_ is comparable to that of Co_3_O_4_ (Table 2), suggesting that morphology alone does not translate into a higher accessible area under synthesis and calcination conditions used in the study.

**Figure 2:**
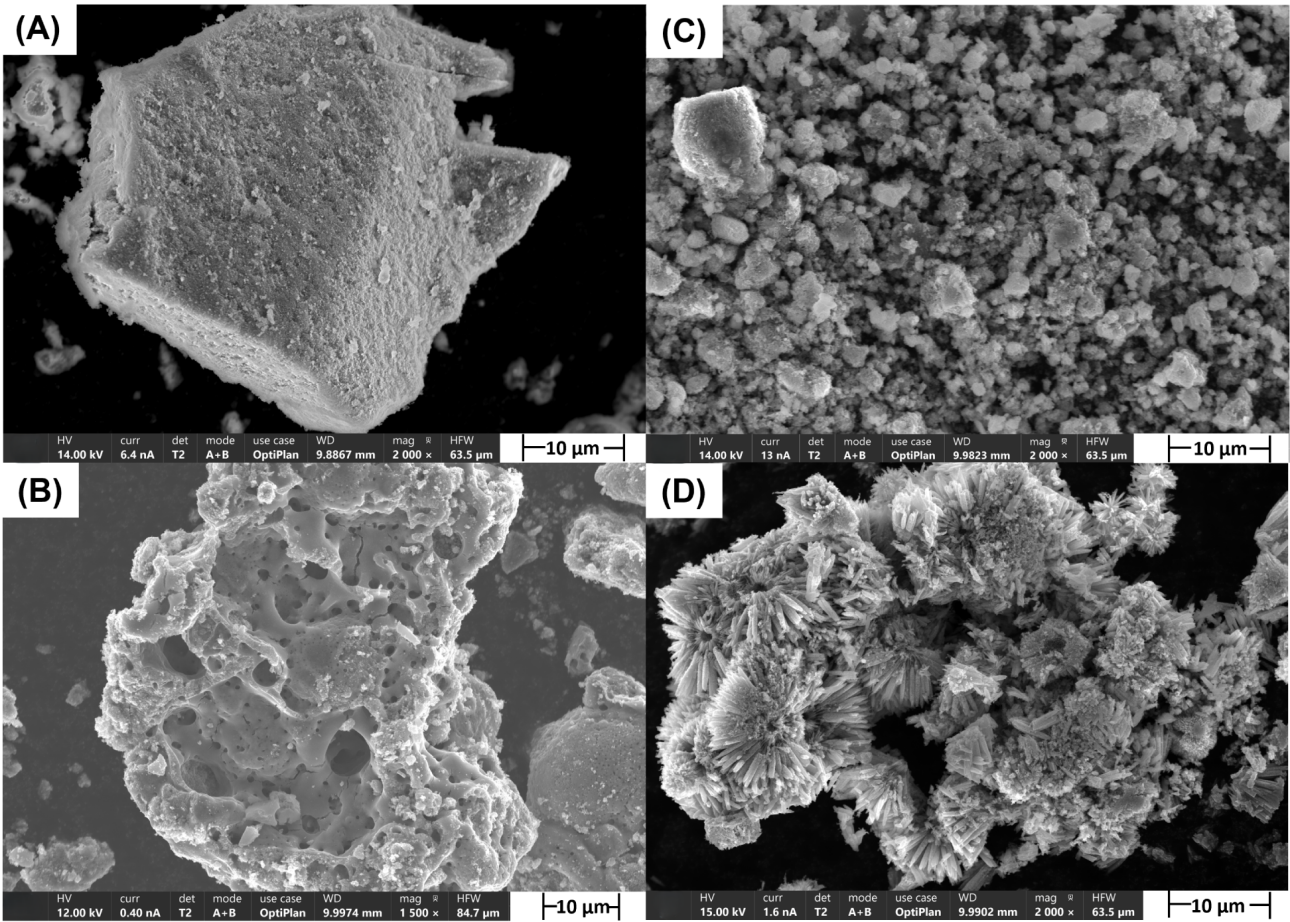
SEM images of transition-metal oxide catalysts: (A) Co_3_O_4_, (B) Mn*_x_* O*_y_*–BaO, (C) Co_3_O_4_–Mn*_x_* O*_y_*, and (D) NiCo_2_O_4_.

**Table 2:** Structural and Surface Information of Prepared Catalysts

	Crystallite size (nm)	BET surface area (m^2^/g)	Average pore diameter (nm)	Average pore volume (cm^3^/g)	(O_II_ + O_III_)/O_I_	Co^3+^/Co^^2+^^	Mn^3+^/Mn^^4+^^	S_basic_/S_acid_
Co_3_O_4_	22.86	23.82	27.94	0.166	1.925	0.326	-	1.340
NiCo_2_O_4_	26.03	21.03	34.78	0.183	1.287	0.466	-	1.132
Co_3_O_4_–Mn_*x*_O_*y*_	9.930	138.6	13.73	0.464	3.697	0.501	0.894	1.577
Mn_*x*_O_*y*_–BaO	33.57	58.85	18.18	0.268	0.780	-	1.493	0.943

The crystalline structures of the catalysts were analyzed using XRD, and the results are shown in Figure 3A. All the peaks in the XRD patterns of the prepared samples are clearly identifiable, confirming the formation of crystalline metal oxides. NiCo_2_O_4_ displayed nearly identical diffraction peaks to Co_3_O_4_, with the exception of an additional peak at 77.2°. This similarity arises from their cubic spinel structure, in which metal cations occupy tetrahedral and octahedral sites in a comparable manner, resulting in similar interplanar spacings (*d*-spacings) and XRD patterns.^31^ In contrast, the diffraction peaks of the Co_3_O_4_–Mn_*x*_O_*y*_ mixture are noticeably attenuated and broadened compared to pure Co_3_O_4_. This peak broadening likely arises from a combination of decreased crystallite size and lattice strain induced by the addition of manganese. The larger ionic radii of Mn^2+^ and Mn^3+^ may distort the Co_3_O_4_ lattice, which can replace tetrahedrally coordinated Co^2+^ and octahedrally coordinated Co^3+^. Consistent with this, the Scherrer equation calculations reveal that Mn_*x*_O_*y*_ incorporation limits crystallite growth, further contributing to peak broadening due to increased strain (Table 2).^32^ The reduction in crystallite size is generally associated with an increase in surface area, which enhances catalytic performance.^33^ This improvement is due to the presence of amorphous regions that create additional interfaces and defect sites, thereby increasing the concentration of oxygen vacancies and promoting methane activation.^34^ In the case of the Mn_*x*_O_*y*_–BaO mixture, the XRD pattern also shows broad peaks and low crystallinity due to lattice strain caused by the incorporation of Ba, which has a larger ionic radius. Additionally, diffraction peaks confirmed the presence of Mn_2_O_4_ and BaMnO_4_ compounds.

**Figure 3:**
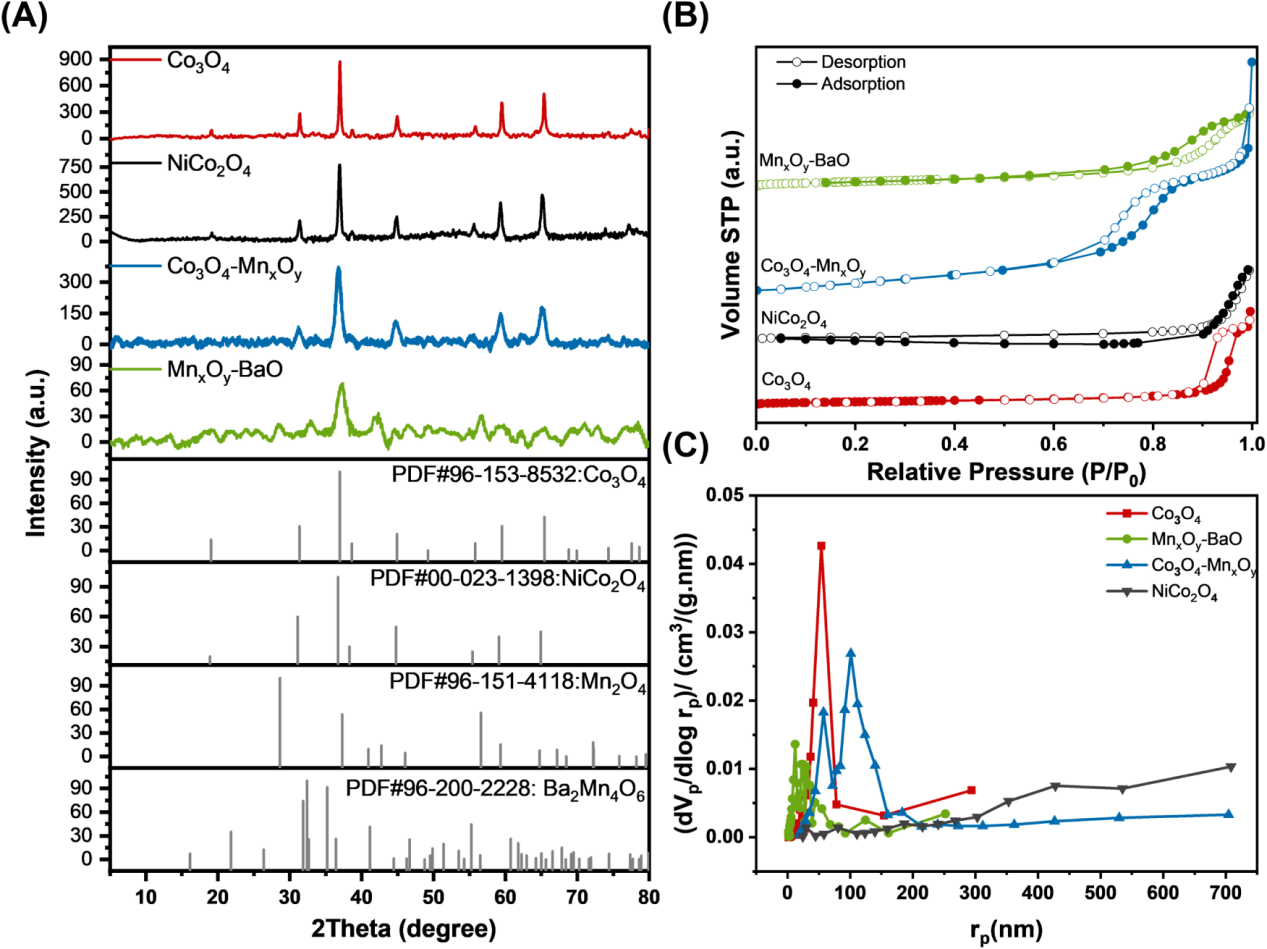
(A) XRD diffraction peaks of the catalysts. (B) N_2_ adsorption–desorption isotherms. (C) BJH pore-size distributions.

To gain further insights into the structural properties of the catalysts, nitrogen adsorption–desorption measurements were conducted to assess their specific surface areas, pore volumes, and pore size distributions. As shown in Figure 3B, all the metal oxides exhibited type IV isotherms with H3-shaped hysteresis loops, characteristic of nonuniform porous materials that likely contain slit-shaped mesopores or are composed of plate-like particles.^35^ These results indicate that the introduction of different metal oxides does not alter the isotherm shape. Notably, the mixed metal oxides demonstrated significantly higher BET surface areas compared to the pristine samples. For example, the Co_3_O_4_–Mn_*x*_O_*y*_ mixture had a surface area nearly 5–6 times larger than that of pure Co_3_O_4_. Additionally, the position of the hysteresis loop shifted toward higher relative pressures (*P*/*P*_0_) from the Co_3_O_4_–Mn_*x*_O_*y*_ mixture to Co_3_O_4_, indicating an increase in pore size. Specifically, the average pore diameter increased from 13.73 to 27.94 nm, while the pore volume decreased from 0.4635 to 0.166 cm^3^/g (Table 2).

A larger surface area is advantageous for catalytic activity, as it allows the catalyst to expose more active sites. Likewise, a larger pore volume facilitates faster mass diffusion of reactants and improves the adsorption of reactant molecules, thereby increasing catalytic efficiency. In terms of pore size distribution, Co_3_O_4_ exhibited a dominant mesopore population centered around 54 nm, whereas the Co_3_O_4_–Mn_*x*_O_*y*_ and Mn_*x*_O_*y*_–BaO mixtures exhibited broader distributions, with two peaks at 57 and 101 nm for Co_3_O_4_–Mn_*x*_O_*y*_ and three peaks at 12, 22, and 30 nm for Mn_*x*_O_*y*_–BaO. This indicates the presence of a hierarchical mesoporous structure consisting of interconnected pores of varying sizes, including mesopores and macropores (Figure 3C).^36^ Such hierarchical structures provide larger interfaces, multiple channels, and numerous active sites, all of which minimize flow resistance and enhance contact between the catalyst and gas molecules, ultimately improving overall reaction performance.^37^ Lastly, NiCo_2_O_4_ exhibits small peaks, suggesting that the catalyst has a broader pore size distribution with no dominant pore size range indicative of an undefined porous structure or limited mesoporous and macroporous features.^38^

The NH_3_-TPD and CO_2_-TPD analyses were employed to explore the surface acid–base properties of the catalysts, as these properties directly influence oxygen vacancy formation, which is a critical factor for methane oxidation via the Mars-van Krevelen (MvK) mechanism.^39^ The CO_2_-TPD profiles (Figure 4) showed that all catalysts exhibited two desorption regions corresponding to weak and strong basic sites. The weak basic sites observed at lower temperatures (150–200 °C) are associated with physiosorbed CO_2_, while the slightly higher-temperature regime (200–300 °C) corresponds to chemisorbed CO_2_ on surface hydroxyl groups (OH^–^) and weakly bound oxygen.^40^ The strong basic sites observed in the 400–800 °C range were deconvoluted into several subpeaks, which are attributed to the presence of basic sites with varying strength. These subpeaks arise due to differences in the local coordination environment of oxygen species and metal–oxygen interactions.^33^ Co_3_O_4_ and NiCo_2_O_4_ exhibited comparable CO_2_-TPD profiles (Figure 4A and D) characterized by pronounced strong basic sites with desorption occurring at higher temperatures (750–900 °C for Co_3_O_4_ and 600–800 °C for NiCo_2_O_4_). This similarity is consistent with their spinel-type crystal structure observed by XRD and the comparable BET surface areas of these catalysts. Despite these similarities, the S_basic_/S_acid_ ratio is higher for Co_3_O_4_ (1.340) than for NiCo_2_O_4_ (1.132), indicating differences in the relative balance of surface basic and acidic sites between the two catalysts. In contrast, Co_3_O_4_–Mn_*x*_O_*y*_ displays a higher relative contribution of weak basic sites compared to strong basic sites, as evidenced by the more intense low-temperature desorption features. This behavior can be associated with its substantially higher BET surface area (138.6 m^2^ g^–1^), which provides a larger population of exposed surface oxygen species and hydroxyl groups that contribute predominantly to weakly bound CO_2_ adsorption. Such surface characteristics reflect a more defect-rich and heterogeneous oxygen environment, which is also indicated by the XPS O 1s analysis. For Mn_*x*_O_*y*_–BaO (Figure 4B), the strong basic sites observed in the 400–700 °C range were clearly resolved into multiple subpeaks, indicating the presence of basic sites with different strengths arising from Ba–O and Mn–O coordination environments.

**Figure 4:**
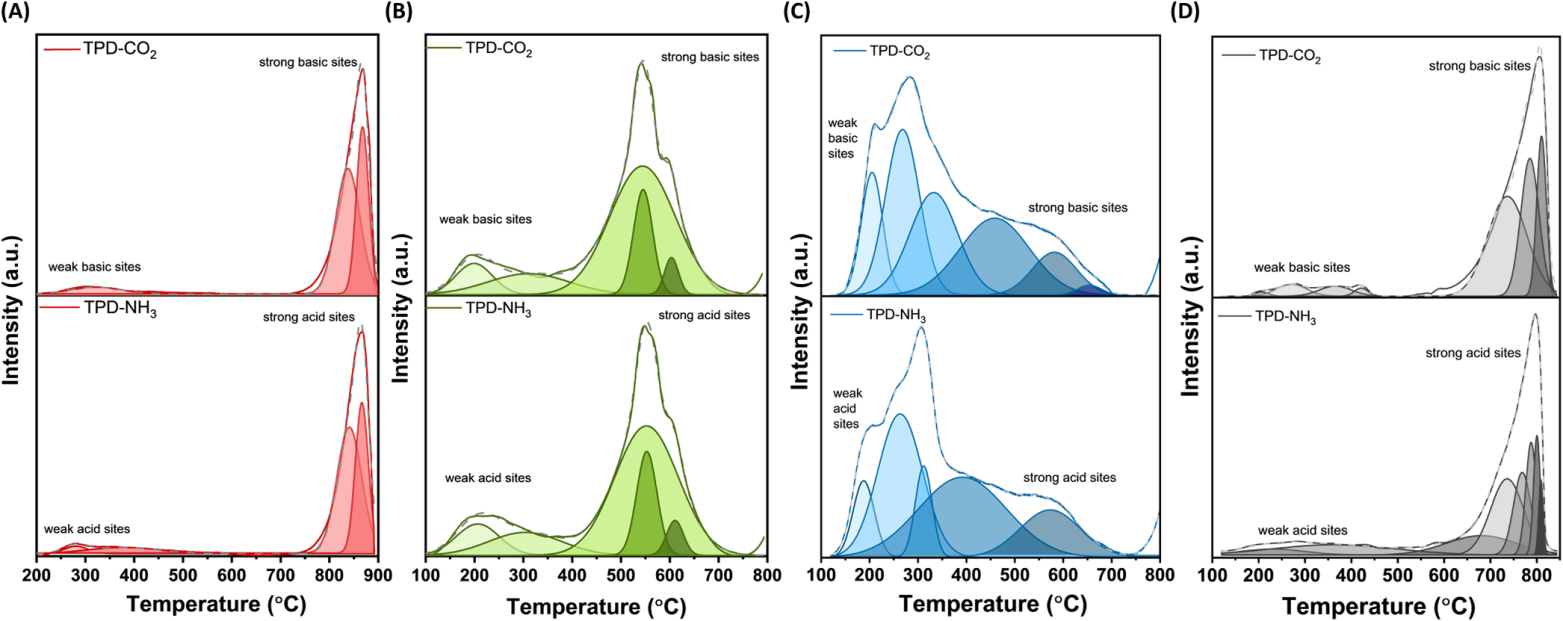
NH_3_-TPD (acidity) and CO_2_-TPD (basicity) profiles for (A) Co_3_O_4_, (B) Mn*_x_* O*_y_*–BaO, (C) Co_3_O_4_–Mn*_x_* O*_y_*, and (D) NiCo_2_O_4_.

For NH_3_-TPD profiles, all catalysts exhibited two main desorption regions, corresponding to the weak and strong acidic sites. In general, the weak acidic sites appeared in the 100–300 °C range and are associated with hydroxylated surface groups or mildly acidic metal–oxygen pairs, while the strong acidic sites were observed between 400 and 800 °C, which are attributed to coordinatively unsaturated metal cations and strongly acidic lattice oxygen species.^41,42^ The S_basic_/S_acid_ ratios vary among the catalysts (Table 2) and follow: Co_3_O_4_–Mn_*x*_O_*y*_ (1.577) > Co_3_O_4_ (1.340) > NiCo_2_O_4_ (1.132) > Mn_*x*_O_*y*_–BaO (0.943), reflecting variations in the balance between surface basic and acidic properties. In the case of NiCo_2_O_4_, the incorporation of Ni into the Co_3_O_4_ spinel lattice may influence surface acidity through changes in cation coordination and local charge balance, which is consistent with its lower S_basic_/S_acid_ ratio and indicates a relatively higher contribution of acidic sites in the Ni-containing spinel.^33^ Mn_*x*_O_*y*_–BaO exhibits the highest acidity among the catalysts according to NH_3_-TPD. Although BaO is intrinsically basic, the combination of BaO with Mn oxides can generate heterogeneous interfacial environments (e.g., Mn–O–Ba linkages) and increase the fraction of coordinatively unsaturated Mn cations at the surface. These under-coordinated metal centers behave as Lewis-acidic sites and therefore contribute to higher NH_3_ uptake.^6,42^

An appropriate balance between surface acidic and basic sites is important for methane activation, as it enables cooperative acid–base interactions at the catalyst surface.^43^ The strong basic sites with oxygen vacancy-related, coordinatively unsaturated oxygen species can promote C–H bond activation and this formation/stabilization of surface-bound reaction intermediates. Simultaneously, the acidic sites can assist in adsorption and polarization of reactants and promote subsequent oxidation steps in conjunction with reactive oxygen species.^13,44^

### Investigation on the Transition Metals and Oxygen Species

The XPS technique was employed to investigate the surface composition and oxidation states of the elements present in the TMO catalyst samples. Regional analysis was conducted for the O 1s, Co 2p, and Mn 2p spectra of all catalysts to identify the chemical states of oxygen, cobalt, and manganese. In addition, the Co 2p and O 1s regions of the Co_3_O_4_ catalyst were analyzed both before and after the reaction to evaluate changes in surface chemistry resulting from the catalytic reaction. As shown in Figure 5A, the deconvolution of the O 1s spectrum reveals the three distinct peaks. The lower binding energy peak, centered around 529 eV, corresponds to lattice oxygen (O_I_) within the crystalline structure of the metal oxides. It is less reactive compared to surface oxygen species but plays an important role in sustaining activity over time by migrating from the bulk to the surface to replenish consumed oxygen during redox cycles. In contrast, the higher binding peaks that are centered around 531–533 eV are attributed to the surface oxygen species and weakly adsorbed oxygen or hydroxyl groups that are found at defects and terminal groups (O_II_ and O_III_).^45^ These surface species are highly reactive and play an important role in the activation of methane via C–H bond cleavage.^46^ Their high reactivity arises from their electron-deficient nature and weak bonding to the catalyst surface, which lowers the energy barrier for hydrogen abstraction and participation in redox cycles.^11,47^ An additional high-binding-energy O 1s component (O_IV_) was observed for Co_3_O_4_ and is attributed to weakly bound surface species such as adsorbed water and/or carbonate species formed upon exposure to ambient conditions. This component was excluded from the quantitative analysis of reactive oxygen species.

**Figure 5:**
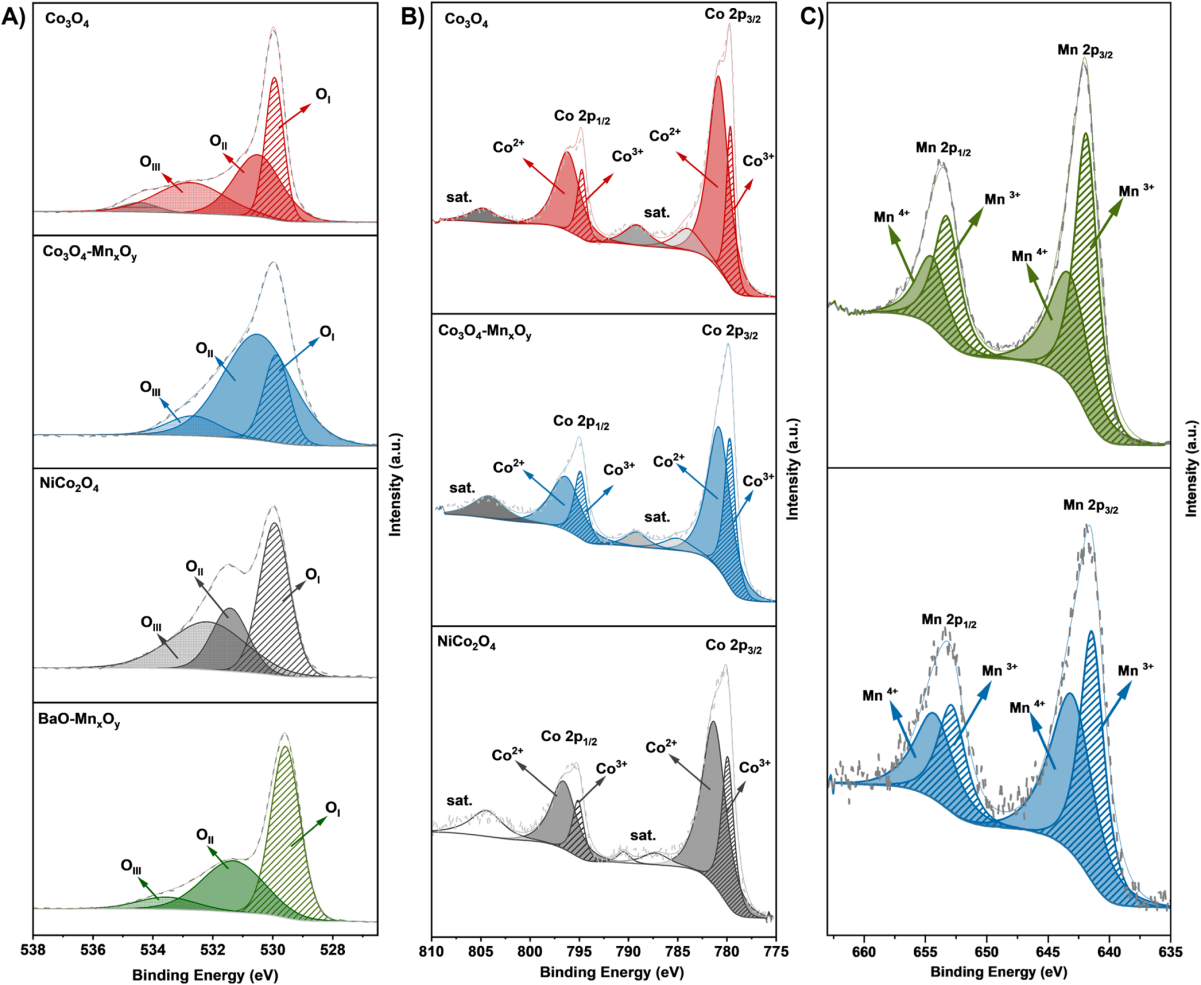
XPS spectra of the catalysts: (A) O 1s spectra, (B) Co 2p spectra, and (C) Mn 2p spectra.

The comparison of the O 1s spectrum for the four catalysts reveals significant differences in the relative proportions of surface and lattice oxygen, which can be directly correlated with their catalytic performance. Co_3_O_4_ and Co_3_O_4_–Mn_*x*_O_*y*_ exhibit the highest (O_II_ + O_III_)/O_I_ ratios of 1.925 and 3.698, respectively, reflecting a substantial contribution of defect-related and surface oxygen species. This is further corroborated by CO_2_-TPD results, where Co_3_O_4_–Mn_*x*_O_*y*_ shows a more pronounced low-temperature desorption peak attributed to weakly basic sites associated with surface oxygen species (O_II_/O_III_), which are active in CO_2_ adsorption/activation. The high-temperature peak corresponds to strong basicity from lattice oxygen (O_I_), consistent with its role in stability. The higher S_basic_/S_acid_ ratios observed for Co_3_O_4_ (1.340) and Co_3_O_4_–Mn_*x*_O_*y*_ (1.577) align with the abundance of reactive surface oxygen. These results agree with the observed catalytic performance, as both catalysts exhibited lower T_50_ values than the other catalysts, indicating higher activity at lower temperatures. In contrast, the O 1s spectrum of Mn_*x*_O_*y*_–BaO reveals a low (O_II_ + O_III_)/O_I_ ratio (0.78), indicating limited abundance of reactive surface oxygen species. This, in conjunction with the lower basicity/acidity ratio (0.943) obtained from TPD, suggests that the introduction of BaO affects the reducibility. Indeed, it has been reported that the BaO introduction decreases the reducibility of manganese and shifts the oxygen distribution toward stable bulk-like species rather than reactive surface oxygen.^48^

The O 1s spectra of Co_3_O_4_ catalyst after the reaction show depletion rates across oxygen species: hydroxyl species (O_II_) decreased by 22% and weakly bound oxygen (O_III_) by 24%, while lattice oxygen (O_I_) showed 2.6% depletion (Figure 6A). This progression corroborates their distinct mechanistic roles in the MvK cycle. The dominant O_II_ consumption suggests that it is the primary reaction initiator, where surface hydroxyl groups facilitate methane activation through rapid hydrogen abstraction. The depletion of O_III_ reflects its important role in catalytic oxidation, as it not only contributes to the complete conversion of intermediates to CO_2_ but also undergoes partial regeneration through the adsorption of gas-phase O_2_. Next, the smaller decrease in O_I_ suggests slower participation in the reaction, reflecting its role as a reservoir for oxygen replenishment to sustain catalytic activity over time. This balance is important: although O_I_ participates in the MvK cycle, its strong lattice binding makes it kinetically slower than surface oxygen processes. Thus, a system where (O_II_ + O_III_) > O_I_ achieves complete oxidation by favoring faster surface-mediated pathways, while still retaining O_I_’s stabilizing function for sustained activity.^49^ While our ex situ XPS captures the endpoint, analogous in situ AP-XPS studies with mixed-metal oxides reported in the literature^50^ demonstrated that the O 1s shoulder peak (∼531–533 eV), which is assigned to surface hydroxyl groups, diminishes as temperature increases, confirming that surface oxygen consumption dominates at lower temperatures. Additionally, by quantifying the surface atomic ratio of oxygen as a function of reaction temperature, it was suggested that oxygen molecules dissociate faster to refill the vacancies, with the refilling process occurring more quickly than the vacancy generation.^50^

**Figure 6:**
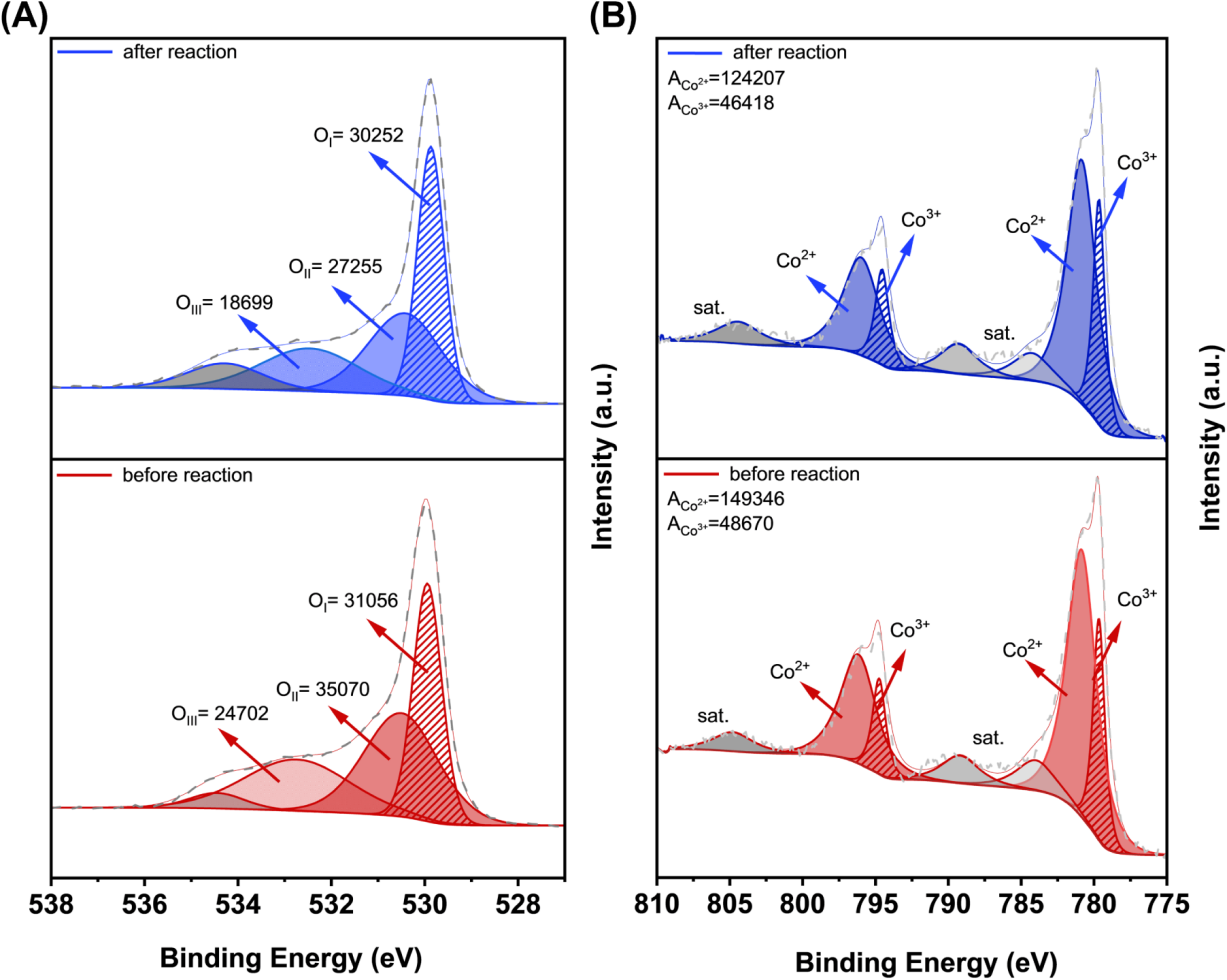
XPS before and after reaction: (A) O 1s spectra and (B) Co 2p spectra.

The Co 2p XPS spectra of three cobalt-containing catalysts shown in Figure 5B exhibit two distinct peaks at 780 and 795 eV, corresponding to Co 2p_3/2_ and Co 2p_1/2_ spin–orbit components, along with additional peaks attributed to shakeup satellites.^51^ Each peak is deconvoluted into octahedral coordination of Co^3+^ (779 and 794–795 eV) and tetrahedral coordination of Co^2+^ (781 and 796 eV) oxidation states.^52,53^ Co^3+^ primarily functions as the oxidizing species responsible for methane activation and oxidation, while Co^2+^ plays a key role in redox cycling and maintaining the structural stability of the catalyst.^54^ The Co^3+^/Co^2+^ ratios derived from the spectra for Co_3_O_4_, Co_3_O_4_–Mn_*x*_O_*y*_, and NiCo_2_O_4_ are 0.326, 0.501, and 0.466, respectively. The increase in this ratio with the incorporation of other transition metals underscores their influence on cobalt’s electronic environment. Nickel contributes through its electron-donating effect, partially stabilizing Co^3+^, though strong Co^3+^–O^2–^ covalent bonding ultimately limits oxygen mobility.^55^ However, despite its high Co^3+^/Co^2+^ ratio (0.466), NiCo_2_O_4_ exhibits lower catalytic activity than other catalysts. Additionally, the large satellite peaks observed in the Co 2p spectrum suggest pronounced charge-transfer effects or complex electronic interactions, which hinder redox activity.^56^ In contrast, Co_3_O_4_–Mn_*x*_O_*y*_ exhibits the highest Co^3+^/Co^2+^ ratio (0.501), which can be attributed to the role of manganese (Mn^3+^ and Mn^4+^) as an electron acceptor.

The Mn 2p XPS spectra (Figure 5C) further confirm the presence of both Mn^3+^ (641 and 653 eV) and Mn^4+^ (643 and 654 eV) oxidation states.^57^ The calculated Mn^3+^/Mn^4+^ ratios differ between manganese-containing catalysts (Table 2). While Mn^3+^ is often associated with enhanced catalytic activity due to oxygen vacancy formation,^52,58^ Mn^3+^/ Mn^4+^ alone does not predict activity, indicating the important role of other factors such as redox coupling and acid–base balance.

The Co 2p XPS spectra of Co_3_O_4_ before and after the reaction reveal notable changes in the oxidation states of cobalt (Figure 6B). After the reaction, the Co^3+^/Co^2+^ ratio increases from 0.326 to 0.374, and this shift reflects the oxidation of Co^2+^ to Co^3+^, which is consistent with the redox requirements of the catalytic process.^59^ In addition, the formation of oxygen vacancies enhances the electron transfer processes within the cobalt oxide lattice, which in turn leads to charge-transfer effects that are reflected in the increased satellite peak area.

### Probable Reaction Mechanism and Dominant Factors for Catalytic Performance

The oxidation of methane over the studied catalysts, including Co_3_O_4_, Co_3_O_4_–Mn_*x*_O_*y*_, Mn_*x*_O_*y*_–BaO, and NiCo_2_O_4_, follows distinct reaction pathways influenced by the interplay of oxygen availability, redox activity, and acid–base properties. Methane interacts with acid–base pairs on the catalyst surface, where its C–H bond undergoes heterolytic cleavage facilitated by metal–oxygen pairs (Mn^3+^/Mn^4+^–O^2–^ in Mn-containing catalysts, Co^3+^–O^2–^ in Co_3_O_4_ and NiCo_2_O_4_ with Ni^2+^ playing a secondary role in modulating oxygen mobility), forming CH_3_^–^ and H^+^ species. This activation is facilitated by reactive oxygen species (O^–^ or O_2_^2–^, denoted as O_II_/O_III_) present on the catalyst surface.^51^

In Co_3_O_4_ and Co_3_O_4_–Mn_*x*_O_*y*_, these reactive surface oxygen species (O_II_ and O_III_) are consumed during methane activation, reacting with intermediates to form CO_2_ and H_2_O as evidenced by their decreased proportion in postreaction XPS analysis. The depletion of surface oxygen is counterbalanced by the migration of lattice oxygen (O_I_), sustaining the redox cycle. The Co^3+^/Co^2+^ratio increases because of the reaction, indicating that Co^2+^ oxidation occurs (Co^2+^ + 1/2 O_2_→ Co^3+^ + O^2–^) to regenerate active sites. Additionally, Co_3_O_4_ and Co_3_O_4_–Mn_*x*_O_*y*_ exhibit a high S_basic_/S_acid_ ratio, which further enhances their catalytic performance by providing a balanced environment for methane activation. The coexistence of Lewis acidic Co^3+^/Co^2+^ sites and adjacent basic oxygen species can cooperatively promote surface-mediated C–H bond reaction activation and stabilize surface-bound intermediates, facilitating subsequent oxidation steps. Also, in Co_3_O_4_–Mn_*x*_O_*y*_, Mn^4+^ promotes Co^2+^ oxidation (Mn^4+^ + Co^2+^ → Mn^3+^ + Co^3+^), creating additional oxygen vacancies. Manganese substitution lowers the oxygen vacancy formation energy and the barrier for oxygen migration, thereby accelerating replenishment of reactive oxygen species on Co_3_O_4_–Mn_*x*_O_*y*_.^34,60^ The Mn^3+^/Mn^4+^ redox pair further aids in stabilizing reactive intermediates and concurrently enhances oxygen mobility. Although the overall apparent activation energy for methane oxidation increases compared to Co_3_O_4_, this is compensated by the significantly higher pre-exponential factor. This indicates that while the apparent barrier of the rate-controlling step is slightly higher on the Co–Mn surface, the increase in active site abundance and the improved oxygen replenishment dynamics allow the composite to achieve superior light-off performance. Consistent with these points, Co_3_O_4_–Mn_*x*_O_*y*_ shows a higher (O_II_ + O_III_)/O_I_ ratio than Co_3_O_4_ (3.697 vs 1.925), and Co_3_O_4_–Mn_*x*_O_*y*_ displays a more intense low-temperature CO_2_-TPD desorption peak assigned to weakly basic O_II_ + O_III_ sites, indicating enhanced surface oxygen availability and mobility.

NiCo_2_O_4_, while following a similar mechanism,^61^ shows distinct behavior due to the presence of Ni^2+^ (Figure S2). XPS analysis reveals a moderately high Co^3+^/Co^2+^ ratio in NiCo_2_O_4_, but it can be attributed to the strong Co^3+^–O^2–^ binding, as indicated by its higher binding energy in the Co 2p XPS spectra. These interactions reduce oxygen mobility, limiting the replenishment of reactive oxygen species and containing the redox cycle. For Mn_*x*_O_*y*_–BaO, the Mn^3+^/Mn^4+^ redox pairs maintain oxygen mobility, but the basicity of Ba^2+^ shifts the oxygen balance toward bulk-like lattice oxygen (O_I_), as observed in the O 1s spectra, reducing the availability of reactive surface oxygen species.

## Conclusions

This study systematically compared four distinct bulk TMO catalyst motifs for low-concentration methane oxidation, providing a comparative framework for structure–activity relationships. The results demonstrate that Co_3_O_4_–Mn_*x*_O_*y*_ and Co_3_O_4_ are promising catalysts, achieving 90% methane conversion at approximately 330 and 380 °C, respectively. Importantly, the observed performance results cannot be attributed to one parameter alone but rather to the interplay of structural, textural, and surface-chemical properties. The enhanced catalytic performance of Co_3_O_4_–Mn_*x*_O_*y*_ could be attributed to its morphological advantages (small crystallite size (9.93 nm) and the large specific surface area (138.6 m^2^/g)), which increase the exposure of active sites and the availability of active oxygen species. In contrast, Co_3_O_4_ exhibits intrinsic advantages due to its high density of surface oxygen vacancies, optimal redox cycling, and balanced surface acidic-basic sites. Notably, Co_3_O_4_–Mn_*x*_O_*y*_ demonstrates competitive light-off behavior among bulk TMO catalysts tested in this study (Table S1), emphasizing its significant potential for practical applications.

Methane oxidation over these catalysts proceeds through a mechanism in which surface oxygen species play a key role in methane activation, cleaving the strong C–H bond. The consumption of reactive surface oxygen is replenished by lattice oxygen, which migrates to the surface through the formation of oxygen vacancies. This dynamic interplay ensures sustained catalytic activity and highlights the critical role of oxygen mobility, redox cycling, and the accessibility of active sites in determining the catalyst’s performance.

These findings provide insights into the rational design of TMO-based catalysts, presenting pathways toward cost-effective and environmentally friendly solutions for low-temperature methane oxidation. The result of this study holds promise for addressing methane emissions from diverse sources, including the significant methane generated by livestock and manure management on agricultural farms, natural sources like wetlands, and even the challenging removal of methane from ambient air. By deepening understanding of methane oxidation mechanisms and catalyst design, this work connects fundamental science and practical applications, enabling targeted solutions for reducing methane emissions and mitigating climate change.

## Supplementary Material



## Notes

Schematic illustrations of the experimental setup and details about XPS spectra fitting information and statistics (PDF)
