# Supplementary material for: Transition Metal-Based Oxidation Catalysts to Mitigate Methane Emissions from Low-Concentration Sources
Source: ACS Omega. 2026 Apr 28;11(18):26220–32. doi: 10.1021/acsomega.5c10282 (PMC13177228; doi:10.1021/acsomega.5c10282)
Supplement: Supplementary file 1 [file ao5c10282_si_001.pdf]

Transition Metal-Based Oxidation Catalysts to Mitigate Methane Emissions from Low-Concentration Sources

*Nardana Bazybek[1], Luigi Vicidomini[1,2], Efthymios Kantarelis[1], Klas Engvall[1], Shareq Mohd Nazir[1] **

1. KTH Royal Institute of Technology, Department of Chemical Engineering, Stockholm, SE 114 28, Sweden.

2. Politecnico Milano, Department of Chemical Engineering, Milan, IT 20133, Italy.

Supporting information

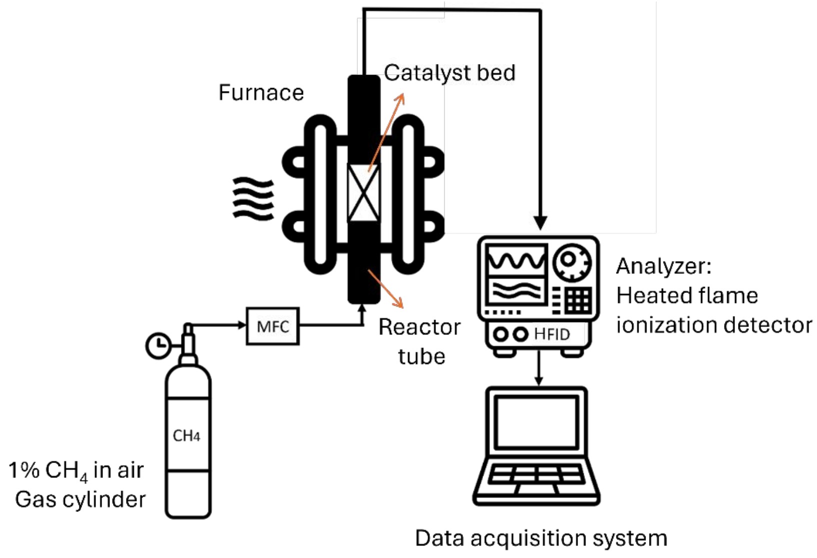

Figure S1. Experimental setup

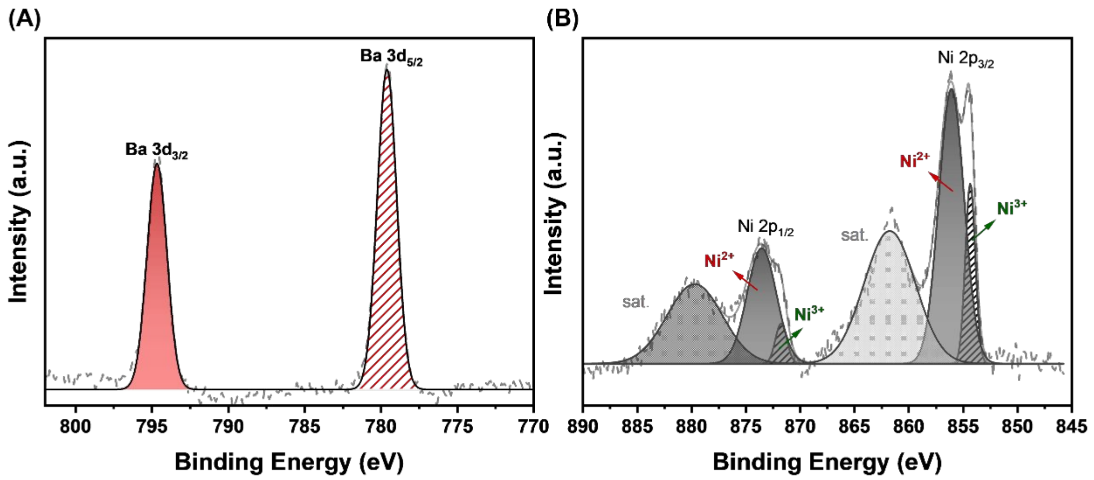

Figure S2. (A) Ba 3d spectra and (B) Ni 2p spectra of transition metal oxides.

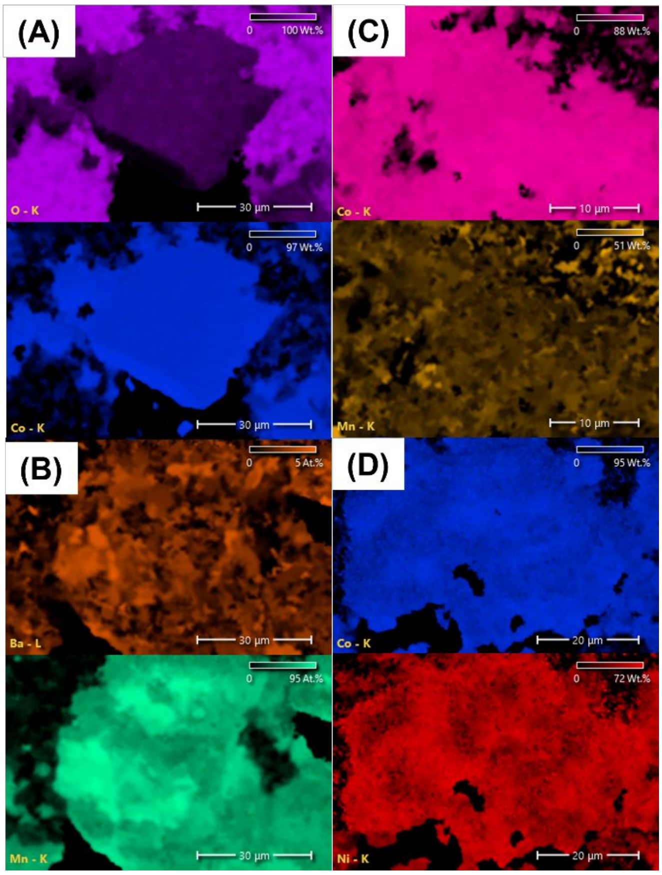

Figure S3. EDS images and corresponding EDS elemental mapping of transition-metal oxide catalysts: (A) Co_3O_4, (B) Mn_xO_y-BaO, (C) Co_3O_4-Mn_xO_y and (D) $NiCo_2O_4$.

Table S1. Catalytic activity comparison between literature-reported catalysts and the present work

Catalysts	Feed conditions	GHSV ($mL{\cdot}g^{-1}{\cdot}h^{-1}$)	T_{50} (˚C)	T_{90} (˚C)	Reference
NiO-PEG	1% CH_4, 22% O_2, balanced in N_2	18000	370	400	[1]
$NiCo_2O_4$	5% CH_4, 25% O_2 and 70% Ar	24000	275	300	[2]
$NiCo_2O_4$ bubbles	1% CH_4, 20% O_2, balanced in N_2	30000	346	401	[3]
MB-Co_3O_4	1% CH_4, 20% O_2, balanced in N_2	30000	355	454	[3]
Co_3O_4	1% CH_4, 5% O_2, and balanced in N_2	50000	350	425	[4]

Co_3O_4	1% CH_4, 20% O_2, balanced in N_2	60000	374	445	[5]
$Ni_{0.5}Co_{2.5}O_4$	1% CH_4, 20% O_2, balanced in N_2	60000	374	445	[5]
Pd(PdO)/Co_3O_4@SiO_2	1% CH_4, 21% O_2, balanced in N_2	30000	357	445	[6]
MnO_2-C	-	-	293	350	[7]
CuO(10)-MnO_x	O_2/ CH_4= 6:1, balanced in Ar	24000	355	405	[8]
$CoMnO_x$	1% CH_4, 20%O_2, balanced in N_2	24000	285	340	[9]
Co_3O_4	1% CH_4, 20%O_2, balanced in N_2	24000	332	380	This work
Mn_xO_y-BaO	1% CH_4, 20%O_2, balanced in N_2	24000	372	440	This work
Co_3O_4-Mn_xO_y	1% CH_4, 20%O_2, balanced in N_2	24000	314	330	This work
$NiCo_2O_4$	1% CH_4, 20%O_2, balanced in N_2	24000	436	475	This work

Table S2. Elemental composition of the catalysts according to the EDS mapping.

Catalyst	Element	Atomic %	Atomic % Error	Weight %	Weight % Error
**Mn_xO_y-BaO**	O	47.7	0.2	19.9	0.1
	Mn	49.9	0.1	71.4	0.1
	Ba	2.5	0.0	8.8	0.0
**Co_3O_4**	O	51.1	0.2	22.1	0.1
	Co	48.9	0.1	77.9	0.2
**Co_3O_4-Mn_xO_y**	O	37.5	0.2	14.2	0.1
	Mn	11.9	0.0	15.5	0.1

Catalyst	Element	Atomic %	Atomic % Error	Weight %	Weight % Error
	Co	50.6	0.1	70.4	0.2
$NiCo_2O_4$	O	34.9	0.2	12.7	0.1
	Co	45.6	0.1	61.2	0.1
	Ni	19.5	0.1	26.1	0.1

Table S3. O 1s peak fitting parameters

Catalyst	Peak	Position (eV)	FWHM	Area	%Area	Residual STD
Co_3O_4	OI	529.94	0.72	31 056.33	32.8	1.151
	OII	530.50	1.87	35 070.04	37.04	
	OIII	532.74	2.66	24 702.12	26.09	
	OIV	534.45	1.37	3853.84	4.07	
Co_3O_4-Mn_xO_y	OI	529.87	1.01	20 471.72	21.29	1.095
	OII	530.5	2.70	66 491.21	69.14	
	OIII	532.66	2.01	9 202.27	9.57	
$NiCo_2O_4$	OI	529.94	1.19	38 069.39	43.73	0.987
	OII	531.42	1.43	18 901.74	21.71	
	OIII	532.17	2.91	30 084.25	34.56	
Mn_xO_y-BaO	OI	529.58	1.14	50 219.70	56.19	1.134
	OII	531.32	2.44	32 018.41	35.82	
	OIII	533.54	2.26	7 141.93	7.99	

Table S4. Co 2p peak fitting parameters

Catalyst	Peak	Position (eV)	FWHM	Area	%Area	Residual STD
$NiCo_2O_4$	Co $2p_{3/2}$ 3^+	779.91	1.41	10 888.57	18.41	1.452
	Co $2p_{3/2}$ 2^+	781.29	2.72	23 385.43	39.54	
	Sat I	787.00	3.14	2 090.90	3.54	
	Sat II	790.46	1.30	739.97	1.25	
	Co $2p_{1/2}$ 3^+	794.91	1.66	5 444.29	9.20	
	Co $2p_{1/2}$ 2^+	796.29	3.50	11 692.71	19.77	
	Sat III	804.67	3.83	4 904.35	8.29	
Co_3O_4	Co $2p_{3/2}$ 3^+	779.66	0.93	32 446.58	13.76	1.298
	Co $2p_{3/2}$ 2^+	781.17	2.37	99 564.86	42.22	
	Sat I	784.00	3.11	14 788.72	6.27	

	Sat II	789.18	3.05	13 299.38	5.64	
	Co $2p_{1/2}$ 3^{+}	794.65	1.08	16 223.29	6.88	
	Co $2p_{1/2}$ 2^{+}	796.17	2.90	49 782.43	21.11	
	Sat III	804.78	3.25	9 729.75	4.13	
**Co_3O_4-Mn_xO_y**	Co $2p_{3/2}$ 3^{+}	779.71	1.31	19 459.63	18.71	1.396
	Co $2p_{3/2}$ 2^{+}	781.30	2.72	38 848.35	37.36	
	Sat I	785.07	3.28	3 973.94	3.82	
	Sat II	789.20	2.61	3 846.29	3.70	
	Co $2p_{1/2}$ 3^{+}	794.70	1.45	9 729.81	9.36	
	Co $2p_{1/2}$ 2^{+}	796.35	3.64	19 424.18	18.68	
	Sat III	804.19	3.89	8 707.92	8.37	

Table S5. Mn 2p peak fitting parameters

Catalyst	Peak	Position (eV)	FWHM	Area	%Area	Residual STD
**Co_3O_4-Mn_xO_y**	Mn $2p_{3/2}$ 3^{+}	641.25	2.31	7 546.94	31.45	1.344
	Mn $2p_{3/2}$ 4^{+}	643.04	3.98	8 449.67	35.21	
	Mn $2p_{1/2}$ 3^{+}	652.85	2.70	3 773.47	15.73	
	Mn $2p_{1/2}$ 4^{+}	654.66	3.76	4 224.83	17.61	
**Mn_xO_y-BaO**	Mn $2p_{3/2}$ 3^{+}	641.87	2.18	56 412.28	39.83	1.578
	Mn $2p_{3/2}$ 4^{+}	643.28	3.75	38 013.48	26.84	
	Mn $2p_{1/2}$ 3^{+}	653.45	2.45	28 206.14	19.91	
	Mn $2p_{1/2}$ 4^{+}	654.88	2.95	19 006.74	13.42	

Table S6. Co 2p and O1s peak fitting parameters for the Co_3O_4 after the reaction.

Core level	Peak	Position (eV)	FWHM	Area	%Area	Residual STD
**Co 2p**	Co $2p_{3/2}$ 3^{+}	779.72	0.93	32 808.69	15.61	1.360
	Co $2p_{3/2}$ 2^{+}	780.92	2.35	84 920.77	40.41	
	Sat I	783.67	3.04	13 973.04	6.65	
	Sat II	789.24	2.98	14 294.59	6.80	
	Co $2p_{1/2}$ 3^{+}	794.72	0.96	13 610.14	6.48	
	Co $2p_{1/2}$ 2^{+}	795.92	2.60	39 287.53	18.69	
	Sat III	804.75	3.30	11 256.86	5.36	
**O 1s**	OI	529.95	0.71	30 252.12	35.68	0.970
	OII	530.52	1.96	27 255.34	32.15	
	OIII	532.71	2.59	18 698.70	22.06	
	OIV	534.61	2.00	8 573.27	10.11	

Peak fitting for XPS

High-resolution spectra were processed and fitted using CasaXPS software (version 2.3.27). Relative sensitivity factors (RSFs) for the corresponding X-ray source were taken from the Kratos library to account for the analyzer transmission function and photoionization cross sections. A Shirley-type background was applied consistently to all core-level spectra.

Peak fitting was carried out using mixed Gaussian–Lorentzian line shapes, expressed in CasaXPS notation as GL(X), with the Lorentzian contribution selected according to the intrinsic lifetime broadening of the respective core hole. For transition-metal core levels (Co 2p and Mn 2p), a GL(80) line shape was used, whereas the O 1s region was fitted using a GL(30) profile. The fitting procedure was optimized by minimizing the standard deviation of the residuals. Peak positions and full widths at half maximum (FWHM) were constrained based on established literature values[10].

The Co 2p spectra were fitted by including contributions from Co $2^{+}$and Co 3^{+}species together with their corresponding shake-up satellite features, whose positions and widths were constrained based on established literature reports. The spin-orbit splitting between the Co $2p_{3/2}$ and Co $2p_{1/2}$ components was fixed at approximately 15.0 eV, and the area of each Co $2p_{1/2}$ component was constrained to be half that of the corresponding Co $2p_{3/2}$ peak, in accordance with the expected 2:1 degeneracy of the spin-orbit doublet. Identical fitting constraints were applied to all samples to ensure consistency.

The Mn $2p_{3/2}$ and Mn $2p_{1/2}$ components were fitted with a fixed spin–orbit splitting of approximately 11.6 eV, and the intensity of each Mn $2p_{1/2}$ component was constrained to be half that of the corresponding Mn $2p_{3/2}$ component. Each Mn 2p spin–orbit component was further deconvoluted into contributions from Mn^{3+} and Mn^{4+} oxidation states, with peak positions and full widths at half maximum constrained according to reported reference values for manganese oxides. Identical fitting parameters were applied across all samples to ensure consistent comparison of manganese oxidation states.

The O1s spectra were deconvoluted into three components: a low-binding-energy peak attributed to lattice oxygen (O_{I}) with binding energies in the range of 529.5-530.0 eV; an intermediate-binding-energy component (O_{II}) at 530.0-532.0 eV associated with surface hydroxyl species or defective oxygen; and a higher-binding-energy contribution at 532.0-533.5 eV (O_{III}) corresponding to adsorbed molecular water or carbonate species. The same fitting strategy and line-shape functions were applied to all samples, while the full widths at half maximum of individual components were allowed to vary within physically reasonable limits to reflect differences in surface structure and chemical environment among the catalyst.

References:

(1) Xu, X.; Li, L.; Yu, F.; Peng, H.; Fang, X.; Wang, X. Mesoporous high surface area NiO synthesized with soft templates: Remarkable for catalytic CH4 deep oxidation. *Molecular Catalysis* **2017**, *441*, 81-91. DOI: https://doi.org/10.1016/j.mcat.2017.08.005.
(2) Tao, F. F.; Shan, J. J.; Nguyen, L.; Wang, Z.; Zhang, S.; Zhang, L.; Wu, Z.; Huang, W.; Zeng, S.; Hu, P. Understanding complete oxidation of methane on spinel oxides at a molecular level. *Nature Communications* **2015**, *6*, Article. DOI: 10.1038/ncomms8798 Scopus.
(3) Wen, W.; Che, J.-W.; Wu, J.-M.; Kobayashi, H.; Pan, Y.; Wen, W.; Dai, Y.-H.; Huang, W.; Fu, C.; Zhou, Q.; et al. Co3+–O Bond Elongation Unlocks Co3O4 for Methane Activation under Ambient Conditions. *ACS Catalysis* **2022**, *12* (12), 7037-7045. DOI: 10.1021/acscatal.1c05744.
(4) Xu, H.; Chen, X.; Lin, J.; Zheng, Y.; Xiao, Y.; Zheng, Y. Enhanced Methane Combustion Performance over Zinc-Aided Co3O4 by Engineering the Reactivity of Active Oxygen Species. *Energy & Fuels* **2022**, *36* (21), 13168-13178. DOI: 10.1021/acs.energyfuels.2c02719.
(5) Song, L.; Zhang, H.; Nie, Z.; Tian, J.; Liu, Y.; Ma, C.; Liu, P.; Ren, Q.; Xiong, J.; Huang, H.; et al. Ni Doping Promotes C–H Bond Activation and Conversion of Key Intermediates for Total Oxidation of Methane over Co3O4 Catalysts. *ACS Catalysis* **2023**, *13* (24), 15779-15793. DOI: 10.1021/acscatal.3c03623.
(6) Ma, Y.; Li, S.; Zhang, T.; Zhang, Y.; Wang, X.; Xiao, Y.; Zhan, Y.; Jiang, L. Construction of a Pd(PdO)/Co3O4@SiO2 core–shell structure for efficient low-temperature methane combustion. *Nanoscale* **2021**, *13* (9), 5026-5032, 10.1039/D0NR08723H. DOI: 10.1039/D0NR08723H.
(7) Zhang, K.; Peng, X.; Cao, Y.; Yang, H.; Wang, X.; Zhang, Y.; Zheng, Y.; Xiao, Y.; Jiang, L. Effect of MnO2 morphology on its catalytic performance in lean methane combustion. *Materials Research Bulletin* **2019**, *111*, 338-341. DOI: https://doi.org/10.1016/j.materresbull.2018.11.023.
(8) Akbari, E.; Alavi, S. M.; Rezaei, M.; Montazeri, Z. AOx–MnOx (A = Ni, Cu, Fe, or Co) Nanocatalysts Fabricated by the Mechanochemical Preparation Method for Lean Methane Catalytic Combustion Assisted by a DBD Plasma Reactor. *ACS Applied Nano Materials* **2023**, *6* (18), 16189-16200. DOI: 10.1021/acsanm.3c02129.
(9) Wang, W.; Qiu, R.; Li, C.; Zhong, R.; Wang, H.; Qi, J. Advancing catalytic oxidation of lean methane over cobalt-manganese oxide via a phase-engineered amorphous/crystalline interface. *Chemical Communications* **2024**, *60* (67), 8896-8899, 10.1039/D4CC02696A. DOI: 10.1039/D4CC02696A.
(10) Fantin, R.; Van Roekeghem, A.; Benayad, A. Revisiting Co 2p core-level photoemission in LiCoO2 by in-lab soft and hard X-ray photoelectron spectroscopy: A depth-dependent study of cobalt electronic structure. *Surface and Interface Analysis* **2023**, *55* (6-7), 489-495. DOI: https://doi.org/10.1002/sia.7167. Biesinger, M. C.; Payne, B. P.; Grosvenor, A. P.; Lau, L. W. M.; Gerson, A. R.; Smart, R. S. C. Resolving surface chemical states in XPS analysis of first row transition metals, oxides and hydroxides: Cr, Mn, Fe, Co and Ni. *Applied Surface Science* **2011**, *257* (7), 2717-2730. DOI: https://doi.org/10.1016/j.apsusc.2010.10.051. Yin, J.; Yang, H.; Gan, Z.; Gao, Y.; Feng, X.; Wang, M.; Yang, G.; Cheng, Y.; Xu, X. Electron transmission matrix and anion regulation strategy-derived oxygen-deficient δ-MnO2 for a high-rate and long-life aqueous zinc-ion battery. *Nanoscale* **2023**, *15* (13), 6353-6362, 10.1039/D2NR07282C. DOI: 10.1039/D2NR07282C. *XPS Fitting Guide.*

https://www.xpsfitting.com/ (accessed 2025 November 14). Zhang, S.; Shan, J. J.; Zhu, Y.; Frenkel, A. I.; Patlolla, A.; Huang, W.; Yoon, S. J.; Wang, L.; Yoshida, H.; Takeda, S.; et al. WGS catalysis and in situ studies of CoO(1-x), PtCo(n)/Co3O4, and Pt(m)Co(m')/CoO(1-x) nanorod catalysts. *J Am Chem Soc* **2013**, *135* (22), 8283-8293. DOI: 10.1021/ja401967y From NLM.
